# Embolization of Thyroid Arteries as a Bridge Therapy Before Thyroidectomy in a Patient With Thyrotoxicosis: Case Report

**DOI:** 10.1155/crie/8194736

**Published:** 2026-10-26

**Authors:** Cesar Alfonso Gomez-Fontalvo, Silvia Alejandra Díaz–Jaimes, María Paula Rangel–Villamizar, Diego Augusto Moreno-Diaz, Rafael Castellanos-Bueno

**Affiliations:** ^1^ Department of Internal Medicine Universidad Industrial de Santander Bucaramanga Santander Colombia uis.edu.co

**Keywords:** case report, Graves’ disease, interventional radiology, thyroid artery embolization, thyroid storm

## Abstract

Thyroid artery embolization (TAE) is a minimally invasive technique that induces selective ischemic necrosis of the thyroid parenchyma, thereby reducing thyroid hormone synthesis with few associated complications. Successful cases of TAE as a bridge therapy before total thyroidectomy in patients with refractory thyrotoxicosis are rare. We present the case of a 36‐year‐old man with a history of Graves’ disease and poor adherence to medical therapy who presented with clinical features of thyrotoxicosis. On physical examination, he exhibited active moderate‐to‐severe orbitopathy (EUGOGO classification; clinical activity score 5/7) and a grade III goiter, reflecting advanced and poorly controlled disease. Paraclinical findings revealed markedly elevated free T4 levels and suppressed TSH. The diagnosis of uncontrolled hyperthyroidism was made, with a Burch–Wartofsky score of 40 points, indicating an impending thyroid storm. Medical therapy at maximally tolerated doses did not result in a complete clinical response; therefore, a total thyroidectomy was planned, preceded by therapeutic plasma exchange (TPE). Due to intolerance to TPE, TAE was performed as a bridge therapy, followed by total thyroidectomy without complications. The patient was discharged with a resolution of symptoms and progressive normalization of thyroid hormone levels. TAE may represent a valuable bridging therapy before total thyroidectomy in selected patients with severe thyrotoxicosis due to Graves’ disease who are refractory to or unsuitable for conventional therapies.

## 1. Introduction

Hyperthyroidism is a thyroid gland disorder characterized by an increase in thyroid hormone synthesis and secretion, with a prevalence of 1.3% in the United States, 0.8% in Europe, and 0.7% in Colombia [1, 2]. Its most severe expression is thyroid storm, also known as thyrotoxic crisis, in which an exaggerated exacerbation of thyrotoxic manifestations is accompanied by decompensation of thermoregulatory, cardiovascular, central nervous system, and gastrointestinal–hepatic function. It occurs in approximately 1%–2% of patients hospitalized for thyrotoxicosis and carries a mortality rate that has reached up to 30% in historical series [3–5]. Early identification of severe thyrotoxicosis is therefore critical, as it requires aggressive and urgent therapeutic measures to restore systemic homeostasis and prevent progression to thyroid storm. These measures focus on controlling thyroid hormone levels by blocking their synthesis and release, inhibiting the conversion of T4 to T3, providing supportive care, and addressing the precipitating cause. Management strategies include antithyroid drug therapy that blocks thyroid hormone synthesis; bile acid sequestrants that interrupt enterohepatic circulation of thyroid hormones; iodine‐containing solutions such as Lugol’s solution, which inhibit the release of preformed thyroid hormone from the gland; and cholecystographic agents such as iopanoic acid, which inhibit hormone release through their iodine content and additionally reduce the peripheral conversion of T4 to T3 through deiodinase inhibition. However, when these therapies fail or are contraindicated, a total thyroidectomy may be considered. Preoperatively, clinical stabilization by therapeutic plasma exchange (TPE), which safely removes circulating thyroid hormone from the blood, temporarily reducing its concentration, can be used [6–10].

However, in selected cases, conventional treatments are insufficient, requiring the use of novel therapies such as thyroid artery embolization (TAE), a minimally invasive technique increasingly used in patients with Graves’ disease and thyroid nodular follicular disease [11]. TAE is an endovascular procedure that selectively interrupts blood flow to the thyroid gland, leading to ischemic necrosis of part of the thyroid parenchyma and reducing thyroid hormone synthesis with few associated complications. There are limited reports in the literature of successful TAE as a bridge therapy before total thyroidectomy in patients with refractory thyrotoxicosis and even fewer in the specific setting in which TPE is contraindicated, not tolerated, or not feasible [11–15]. This case supports the feasibility of TAE in a third‐level public hospital in northeastern Colombia.

## 2. Case Presentation

A 36‐year‐old male with a history of Graves’ disease and poor treatment adherence presented with complaints of frontal headache, vomiting episodes, palpitations, subjective fever, fine distal tremors, diplopia, anxiety, irritability, difficulty falling asleep, altered bowel movements, and unintentional weight loss. On admission, blood pressure was 161/90 mmHg, heart rate 138 beats per minute, respiratory rate 22 breaths per minute, temperature 37.0°C, and oxygen saturation 95% on room air. Physical examination revealed sinus tachycardia, active moderate‐to‐severe orbitopathy (EUGOGO classification; clinical activity score 5/7), and grade III goiter. Initial laboratory findings showed markedly elevated free T4 (100.0 pmol/L), suppressed TSH, and hypoalbuminemia, while the remaining laboratory values were within normal limits (Table 1). The patient was diagnosed with uncontrolled hyperthyroidism, with a Burch–Wartofsky score of 40 points, which is consistent with an impending thyroid storm. Medical therapy was initiated with methimazole 20 mg orally every 8 h, cholestyramine 4 g orally every 8 h, and propranolol 60 mg orally every 8 h, and due to the findings of active moderate‐to‐severe orbitopathy, a cycle of methylprednisolone 1 g intravenously for 3 days, followed by oral prednisone, was started (Figure 1). Despite this, thyrotoxicosis persisted, and the endocrinology service recommended total thyroidectomy with preoperative TPE for optimization of the thyroid status before surgery. However, the patient developed intolerance to TPE, characterized by a moderate hypersensitivity reaction including extensive urticaria, angioedema, severe pruritus, and dyspnea, despite appropriate premedication, most likely attributable to the fresh frozen plasma used. TPE with human albumin was not performed due to a nationwide shortage of the product during this period. Consequently, therapy was adjusted to maximally tolerated doses of antithyroid medications (methimazole 20 mg orally every 6 h, drops of Lugol’s solution every 8 h, and cholestyramine 4 g orally every 6 h), and transcatheter arterial embolization was employed as a bridging rescue intervention. TAE was performed as follows: the right common femoral artery was accessed under ultrasound guidance, and a femoral vascular sheath was placed. A vertebral catheter was advanced over a hydrophilic guidewire. A Teflon‐coated guidewire was subsequently used, and 100 mL of contrast medium was injected to perform cervical arteriography. The left superior thyroid artery (STA), originating from the left external carotid artery (ECA), demonstrated a marked vascular supply to the left thyroid lobe. At the level of the left thyrocervical trunk, the ipsilateral inferior thyroid artery (ITA) was identified, providing significant perfusion to both the left thyroid lobe and the inferior pole of the right thyroid lobe. The right STA demonstrated minimal contribution to thyroid vascularization, and catheterization of the right ITA was not feasible. A guiding catheter with a hemostatic valve was then used, followed by selective catheterization of the left STA and ITA using a microcatheter over a 0.014‐inch micro guidewire. Embolization was performed using polyvinyl alcohol (PVA) microparticles (150–250 µm) with the aim of achieving distal occlusion of the thyroid arterial bed. This resulted in complete occlusion of the left STA and ITA, markedly reducing perfusion to the left thyroid lobe and the inferior portion of the right thyroid lobe. A total of 5000 IU of heparin was administered during the procedure, and hemostasis was achieved using a vascular closure device (Figure 2). Free T4 levels prior to TAE were reported to be 100.0 pmol/L. After more than 72 h of postembolization monitoring, during which the previously established antithyroid regimen (methimazole, Lugol’s solution, cholestyramine, and propranolol) was maintained without modification, the patient was considered fit for surgery following anesthesiology evaluation, primarily based on clinical improvement, as evidenced by progressive stabilization of thyrotoxicosis symptoms and a reduction in the Burch–Wartofsky score to 15 points, together with a free T4 level of 95.6 pmol/L. Subsequently, the patient underwent a total thyroidectomy without complications. Free T4 levels 24 h after surgery were 51.5 pmol/L. Histopathological examination confirmed Graves’ disease, with no evidence of parathyroid tissue or malignancy. The patient was discharged asymptomatic, with no signs of hyperthyroidism, no calcium abnormalities (serum calcium: 8.6 mg/dL), and a near‐normal thyroid hormone level (free T4: 25.7 pmol/L). Outpatient levothyroxine replacement therapy was initiated. The clinical and biochemical course from admission to discharge, including the evolution of free T4 and the Burch–Wartofsky score, is summarized in Figure 3.

**Figure 1:**
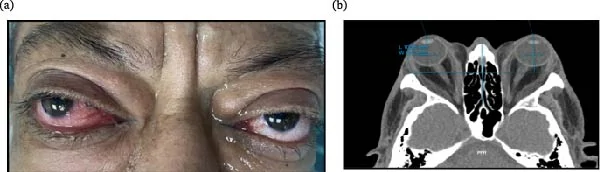
Active moderate‐to‐severe orbitopathy (EUGOGO classification; clinical activity score 5/7): bilateral exophthalmos, lagophthalmos of 7 mm in the right eye and 4 mm in the left eye, bilateral conjunctival hyperemia, caruncular edema, and upper eyelid edema (a). Computed tomography of the orbits: anterior index of 32.9 mm in the right eye and 30.8 mm in the left eye, with bilateral enlargement of the extraocular muscle bellies, most marked in the superior and inferior recti (up to 11 mm) and with relative sparing of the lateral rectus, a pattern characteristic of Graves’ orbitopathy (b). *Source:* authors.

**Figure 2:**
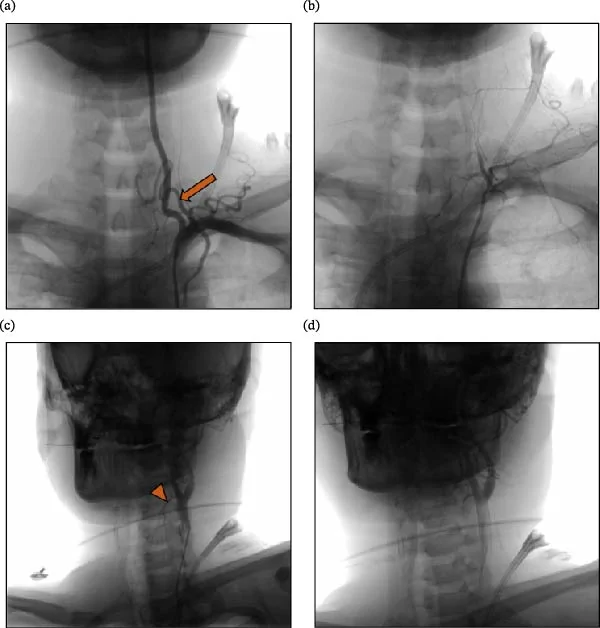
Thyroid artery angiography with selective embolization. (a) The left inferior thyroid artery is indicated by the arrow. (c) The left superior thyroid artery is indicated by the arrowhead. Postembolization of the left superior and inferior thyroid arteries with polyvinyl alcohol microparticle infusion leads to total occlusion (b, d), reducing perfusion to the left thyroid lobe and the lower portion of the right thyroid lobe. The right superior thyroid artery was not embolized due to minimal arterial contribution to the thyroid, as shown in angiography. *Source:* authors.

**Figure 3:**
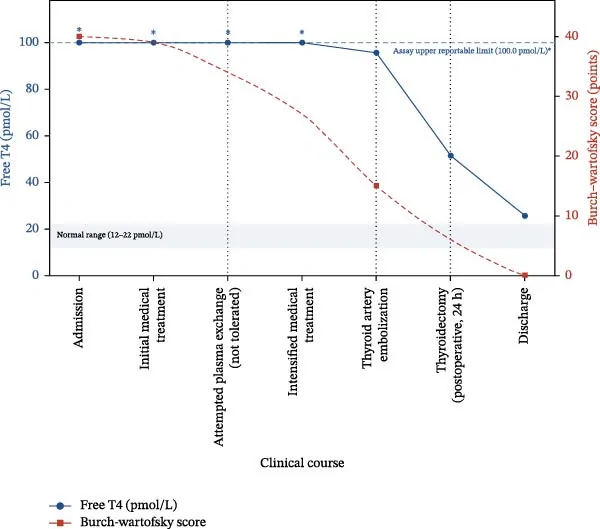
Timeline summarizing the patient’s clinical course, including the biochemical evolution of free T4 and the Burch–Wartofsky score from admission to discharge. TAE, thyroid artery embolization. *Source:* authors.  ^∗^Free T4 values at/above the assay’s upper reportable limit (100.0 pmol/L); the true peak value could not be precisely quantified.

**Table 1:** Initial laboratory findings.

Clinical testing		Admission values	Reference range
Hemogram	Hemoglobin	12.4 g/dL	13–18 g/dL
	Hematocrit	37.5%	42%–52%
	MCV	92.8 fL	86–98 fL
	MCH	29.6 pg	27–32 pg
	MCHC	31.9 g/dL	33–37 g/dL
	Leukocytes	7.13 × 10^3^/µL	5–10 × 10^3^/uL
	Platelets	342 × 10^3^/µL	150–450 × 10^3^/uL
Creatinine		0.31 mg/dL	0.67–1.17 mg/dL
BUN		10.8 mg/dL	6–20 mg/dL
AST		25.4 U/L	6–40 U/L
ALT		25.2 U/L	6–41 U/L
Total bilirubin		0.54 mg/dL	0.1–1.2 mg/dL
Direct bilirubin		0.26 mg/dL	0.1–0.3 mg/dL
PT		14.3 s (INR 1.05)	9.7–13.5 s
aPTT		27.6 s	22.5–30.5 s
LDH		155 U/L	135–250 U/L
Serum potassium		4.23 mmol/L	3.5–4.5 mmol/L
Serum calcium		10.2 mg/dL	8.8–10.2 mg/dL
Albumin		3.13 g/dL	3.97–4.94 g/dL
Alkaline phosphatase		188.0 U/L	40–129 U/L
Free T4		100.0 pmol/L^a^	12–22 pmol/L
TSH		<0.05 µIU/mL	0.27– 4.20 µIU/mL

Abbreviations: ALT, alanine aminotransferase; aPTT, activated partial thromboplastin time; AST, aspartate aminotransferase; BUN, blood urea nitrogen; LDH, lactate dehydrogenase; MCH, mean corpuscular hemoglobin; MCHC, mean corpuscular hemoglobin concentration; MCV, mean corpuscular volume; PT, prothrombin time; TSH, thyroid‐stimulating hormone.

^a^The free T4 assay used at our institution has an upper reportable limit of 100.0 pmol/L.

## 3. Discussion

TAE was first introduced in the 1980s as an alternative therapeutic option for patients at prohibitive surgical risk due to parathyroid neoplasms and selected thyroid malignancies. Its use was later extended to hyperfunctioning thyroid disorders, including nodular follicular thyroid disease (NFTD), autonomously functioning thyroid nodules, and Graves’ disease [12]. In Graves’ disease, TAE has been proposed as an alternative or adjuvant treatment when conventional therapies are ineffective or unavailable; by inducing ischemic necrosis, fibrosis, and apoptosis of the hyperfunctioning parenchyma, it has achieved euthyroid conversion rates exceeding 60%, with durable remission and a progressive decline in TSH receptor antibodies [16, 17]. In thyrotoxicosis, however, evidence is limited to isolated case reports, including thyroid storm and refractory amiodarone‐induced thyrotoxicosis, in which TAE was used as an adjunctive therapy, as an alternative when thyroidectomy was contraindicated, or as a bridge to surgery [11, 13, 16, 17]. More recently, the North American Society for Interventional Thyroidology (NASIT) recognized TAE as an option in selected patients with Graves’ disease when antithyroid drugs fail or are contraindicated and as a preoperative intervention in large, highly vascular goiters, where it may reduce intraoperative blood loss [16]. Nevertheless, given the lack of standardized protocols and the risk of serious complications, its use should be restricted to exceptional circumstances in which standard treatments have failed or are contraindicated and to centers with expertise in embolization [17]. In the present case, TAE was used as a bridge to total thyroidectomy in a patient with Graves’ disease and severe thyrotoxicosis refractory to medical therapy. A comparable strategy was reported by Bonnici et al. [11] in a patient with Graves’ disease and thyroid storm complicated by methimazole‐induced neutropenia, in whom TAE was also successfully performed as a bridge to a total thyroidectomy.

TPE has been increasingly utilized as a bridging strategy in selected patients with severe thyrotoxicosis when conventional therapies are ineffective, contraindicated, or insufficient to achieve rapid biochemical control. TPE may provide temporary biochemical and clinical stabilization through the removal of circulating thyroid hormones, hormone‐binding proteins, proinflammatory cytokines, and thyroid autoantibodies. Recent reports have highlighted its successful use in patients with Graves’ disease complicated by severe hematologic or hepatic adverse effects related to antithyroid drug therapy, facilitating subsequent thyroidectomy or radioiodine treatment following clinical and biochemical stabilization. Nevertheless, the therapeutic effect of TPE is transient due to the continued release of thyroid hormones from intrathyroidal and extrathyroidal stores, underscoring the importance of timely definitive treatment once stabilization is achieved. Despite its recognized role as a bridging therapy, TPE may be limited by procedure‐related complications, patient intolerance, or logistical constraints that hinder its successful completion [10, 18, 19]. In such circumstances, alternative strategies may be required to achieve clinical stabilization prior to definitive treatment as demonstrated in the present case.

Reported complications of TAE are mostly minor, including neck pain, fever, hoarseness, and inguinal hematoma, none of which occurred in our patient. However, serious adverse events have been described in patients treated for hyperthyroidism, including cerebral infarction due to nontarget embolization and death from thyroid crisis, as well as exacerbation of thyroid‐associated orbitopathy in Graves’ disease [17]. Transient hyperthyroidism is expected after TAE due to the release of preformed hormone from the necrotic tissue, peaking within the first 3 days [12, 16]. This highlights the importance of intensive monitoring during the first 72 h postprocedure [11, 12, 14, 15], as well as the need to optimize pharmacological therapy during this period, particularly beta‐blockers such as propranolol and glucocorticoids, both of which inhibit the peripheral conversion of T4 to T3, with glucocorticoids additionally attenuating postembolization inflammation [16]. In our patient, the previously established multimodal pharmacological regimen was maintained without modification at maximally tolerated doses throughout this period; no clinical deterioration occurred, and the active moderate‐to‐severe orbitopathy did not worsen.

A relevant limitation in the interpretation of free T4 values is related to the analytical characteristics of the assay, which has an upper reportable limit of 100.0 pmol/L. Accordingly, in this case, the exact magnitude of free T4 reduction following TAE could not be precisely quantified. This limitation is further compounded by the absence of an established optimal timing for biochemical assessment given the dynamic hormonal changes that may occur during the early postembolization period [14, 15]. Collectively, these factors may have contributed to an underestimation of the true biochemical effect of the procedure. Despite this limitation, free T4 levels decreased below this threshold following the procedure and were accompanied by clinical improvement in thyrotoxicosis, reflected by a reduction in the heart rate from 125 beats per minute before embolization to 90 beats per minute afterwards, a decrease in mean arterial pressure from 109 to 80 mmHg, and resolution of distal tremor and gastrointestinal symptoms. As a result, the patient’s Burch–Wartofsky score fell to 15 points, indicating a low risk of thyroid storm and allowing total thyroidectomy to be performed without complications. This is clinically relevant since thyroidectomy in the setting of uncontrolled hyperthyroidism carries substantial anesthetic and cardiovascular risks. Although the optimal interval between embolization and surgery has not been established [16], in our case, surgery was performed after 72 h, beyond the expected peak of postembolization hormone release. Thus, in this setting, TAE may primarily contribute to enabling definitive surgery rather than achieving biochemical normalization. These findings highlight the need for prospective studies with standardized follow‐up protocols to more rigorously evaluate the clinical efficacy, biochemical impact, and safety profile of TAE in patients with refractory thyrotoxicosis who are not candidates for therapeutic apheresis as a bridge to definitive surgical management.

Given the limited evidence currently available, patient selection remains a critical consideration when contemplating TAE for severe thyrotoxicosis. As discussed previously, TAE has been reported in a variety of complex clinical scenarios, including refractory Graves’ disease, amiodarone‐induced thyrotoxicosis, intolerance or contraindications to conventional antithyroid therapies, and circumstances in which definitive treatment must be facilitated despite limited therapeutic alternatives [11–15]. However, in the absence of standardized indications or evidence‐based patient selection criteria, TAE should not be regarded as a routine therapeutic approach but rather as an individualized rescue or bridging intervention. Consequently, the decision to pursue TAE should be guided by a multidisciplinary assessment that carefully weighs the severity of thyrotoxicosis, the feasibility and limitations of alternative treatment options, the potential risk of procedure‐related complications, including transient worsening of thyrotoxicosis, and the anticipated pathway to definitive therapy. In the present case, persistent severe thyrotoxicosis despite intensive multimodal medical therapy and intolerance to TPE supported the consideration of TAE as a bridge to total thyroidectomy.

Regarding the technical aspects of TAE, it is essential to note that the thyroid gland receives a dual blood supply arising from the STA, originating from the ECA, and the ITA, arising from the STA, originating from the ECA, and the ITA, arising from the thyrocervical trunk [20]. Anatomical variations in arterial origin, acute bifurcation angles, vessel caliber, and/or variable contribution to thyroid vascularization are not uncommon and represent key determinants that interventional radiologists and surgeons must consider when selecting target vessels and assessing the feasibility of selective embolization. Furthermore, standardization of TAE remains challenging due to the marked anatomical variability of the thyroid arterial supply. Consequently, this procedure requires an experienced interventional radiologist capable of meticulous preprocedural planning based on detailed knowledge of these vascular variants, thereby improving the likelihood of successful catheterization of thyroid arterial branches while reducing the risk of nontarget embolization and clinically significant complications [15, 21, 22]. In the present case, embolization of the left STA and ITA resulted in the occlusion of blood flow to the left thyroid lobe and the inferior portion of the right lobe, corresponding to the gland’s predominant vascular supply. Additionally, preservation of at least one ITA is generally recommended to maintain adequate perfusion of the parathyroid glands as both superior and inferior parathyroid glands typically receive their vascular supply from branches of the ITA. Therefore, bilateral occlusion may increase the risk of hypoparathyroidism and subsequent hypocalcemia [23], a risk that may be mitigated by leaving at least one thyroid artery unembolized [16, 17].

## 4. Conclusion

TAE may be considered as an adjunctive bridge therapy before total thyroidectomy in selected patients with severe thyrotoxicosis due to Graves’ disease when conventional medical therapy is insufficient and TPE is not feasible or not tolerated. In the present case, TAE contributed to clinical stabilization within a multimodal treatment strategy, facilitating successful definitive surgical management without procedure‐related complications. However, given the limited evidence currently available, further prospective studies are needed to better define the efficacy, safety, and optimal role of TAE in this setting.

## Funding

This research received no specific funding from public, commercial, or not‐for‐profit agencies.

## Ethics Statement

Written informed consent was obtained from the patient for publication of this case report and the accompanying images, including clinical photographs and radiological images. The patient’s identity was safeguarded. The principles of beneficence and nonmaleficence were adhered to throughout the patient’s treatment.

## Conflicts of Interest

The authors declare no conflicts of interest.

## Data Availability

The data that support the findings of this study are available from the corresponding author upon reasonable request.
